# Mild encephalitis/encephalopathy with reversible splenial lesion (MERS) in adults-a case report and literature review

**DOI:** 10.1186/s12883-017-0875-5

**Published:** 2017-05-25

**Authors:** Junliang Yuan, Shuna Yang, Shuangkun Wang, Wei Qin, Lei Yang, Wenli Hu

**Affiliations:** 1 Department of Neurology, Beijing Chaoyang Hospital, Capital Medical University, Chaoyang District, Beijing, China; 2 Department of Radiology, Beijing Chaoyang Hospital, Capital Medical University, Beijing, China

**Keywords:** Mild encephalitis/encephalopathy with reversible splenial lesion, Adult-onset MERS, Encephalitis, Encephalopathy, Corpus callosum, Reversible plenial lesion

## Abstract

**Background:**

Mild encephalitis/encephalopathy with reversible splenial lesion (MERS) is a rare clinico-radiological entity characterized by the magnetic resonance imaging (MRI) finding of a reversible lesion in the corpus callosum, sometimes involved the symmetrical white matters. Many cases of child-onset MERS with various causes have been reported. However, adult-onset MERS is relatively rare. The clinical characteristics and pathophysiologiccal mechanisms of adult-onset MERS are not well understood. We reviewed the literature on adult-onset MERS in order to describe the characteristics of MERS in adults and to provide experiences for clinician.

**Methods:**

We reported a case of adult-onset MERS with acute urinary retension and performed literature search from PubMed and web of science databases to identify other adult-onset MERS reports from Januarary 2004 to March 2016. Preferred Reporting Items for Systematic Reviews and Meta-Analyses (PRISMA) guideline was followed on selection process. And then we summarized the clinico-radiological features of adult-onset MERS.

**Results:**

Twenty-nine adult-onset MERS cases were reviewed from available literature including the case we have. 86.2% of the cases (25/29) were reported in Asia, especially in Japan. Ages varied between 18 and 59 years old with a 12:17 female-to-male ratio. The major cause was infection by virus or bacteria. Fever and headache were the most common clinical manifestation, and acute urinary retention was observed in 6 patients. All patients recovered completely within a month.

**Conclusion:**

Adult-onset MERS is an entity with a broad clinico-radiological spectrum because of the various diseases and conditions. There are similar characteristics between MERS in adults and children, also some differences.

## Background

Tada et al. first identified the concept of mild encephalitis/encephalopathy with reversible splenial lesion (MERS) as a rare clinico-radiological syndrome in 2004 [1, 2]. In general, patients with MERS presented with mild central nervous system symptoms such as consciousness disturbance, seizures and headache and recovered completely within a month [1, 3]. MERS is divided into two types according to the lesion location. MERS type I, the typical form, most involves a singular lesion in the midline of the splenium of the corpus callosum (SCC), while MERS type II most commonly presents lesions in the symmetrical cerebral white matter or the anterior aspect of the corpus callosum with similar signal manifestations [4, 5]. The typical magnetic resonance imaging (MRI) features are transient high-signal-intensity on T2-weighted images (T2WI), fluid-attenuated inversion recovery images (FLAIR), and diffusion-weighted images (DWI), decreased apparent diffusion coefficient (ADC) value of the lesion on ADC maps, and hyper-isointense signals on T1-weighted imaging (T1WI) sequences without contrast enhancement [1, 4, 6]. Previous studies have identified that MERS can be triggered by infection including influenza virus [7], rotavirus [8], mumps virus [9], Mycoplasma pneumoniae [10] or Legionella pneumophila [11]. In addition to infection, MERS has also been reported to be associated with the administration of antiepileptic drugs [12–14].

Many child-onset MERS cases have been reported, most in Asia, especially Japan [1, 15]. However, adult-onset MERS is relatively rare. Here we reported a case of adult-onset MERS with acute urinary retention. It has been speculated that the characteristics of MERS in adults are different from that in children. So we utilized this opportunity to review the literature on adult-onset MERS in order to describe the clinico-radiological features and establish a clinical position of the disease.

## Methods

### Case presentation

**Fig. 1:**
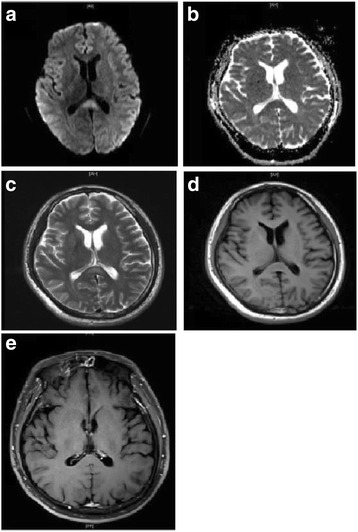
Initial cranial MRI of the patient. The lesion in the midline of SCC was hyperintensity on DWI (**a**) and T2WI (**c**), decreased ADC value (**b**), isointense signals on T1WI (**d**), and no contrast enhancement (**e**)

**Fig. 2:**
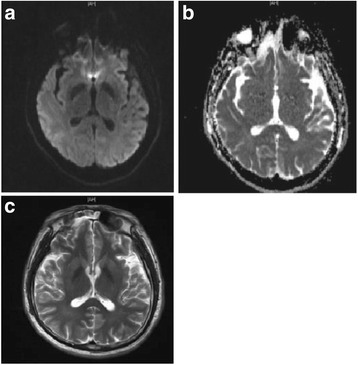
Follow up cranial MRI. The follow up cranial MRI showed no lesion on any sequence (﻿﻿**a**: DWI, **b**:﻿ ADC, **c**: T2WI)

A previously healthy 37-year-old man was admitted to our hospital due to a 9-day history of headache and vomiting. Ten days prior to admission, he suddenly developed a fever of 40 °C, diarrhea and headache. After taking oral antipyretics, he still had a fever of 38–39 °C. Three days before admission, his body temperature returned to normal. Two days before admission, he suffered acute urinary retention and was treated by temporary transurethral catheterization at another hospital. One day before admission, he came to our hospital for acute urinary retention and the catheter was kept. Neurological examination revealed nuchal rigidity positive. Chemistry panel and urine analysis showed no abnormalities except for an elevated blood white cell counts (10.99 × 10^9^/L), C-reactive protein level (9.41 mg/L) and decreased serum sodium (131.8mml/L). Routine immunological screening and tumor markers were negative. Lumbar puncture showed an elevated cerebrospinal fluid (CSF) pressure of 190mmH_2_O. CSF examination demonstrated an increase in white blood cells (97/ul) and protein content (124 mg/dl). The CSF etiological examination was negative. Oligoclonal bands, IgG index and myelin basic protein were within the normal ranges in CSF. Paraneoplastic antibodies were negative. Cranial MRI scans taken on the day after admission showed abnormal signals in SCC, which was hyperintense on T2WI and DWI imaging, decreased on ADC, isointense on T1WI with no contrast enhancement (Fig. 1). The plain and enhancement spinal cord MRI showed no obvious abnormalities. He received intracranial pressure reduction, antiviral, anti-inflammatory and experimental anti-tuberculosis. His urinary retention and fever resolved within 10 days. The follow-up MRI scan taken 14 days after the initial examination showed previous lesion disappeared (Fig. 2). He was discharged home without neurological complications. The final diagnosis was MERS with acute urinary retention.

### Literature search and selection

To better understand the characteristics of adult-onset MERS, we performed a literature search to identify other reports (reviews, case reports or case series) from Januarary 2004 to March 2016, using the PubMed and web of science databases with the following terms, ‘mild encephalitis/encephalopathy with reversible splenial lesion’/‘MERS’/‘reversible splenial lesion’. All pertinent English language articles were retrieved. A hand-search by reviewing the reference sections of the retrieved articles was also performed. The non-English language articles, child-onset MERS reports and not getting full-text articles were excluded. We followed the Preferred Reporting Items for Systematic Reviews and Meta-Analyses (PRISMA) guideline on selection process.

### Data extraction

Two investigators collected data from the selected articles. The following information were extracted: last name of the first author, country where the study was performed, the reported patient’s age, gender, CNS symptoms, neurological examination, etiology, auxiliary examination, therapy and outcome.

## Results of literature review

**Fig. 3:**
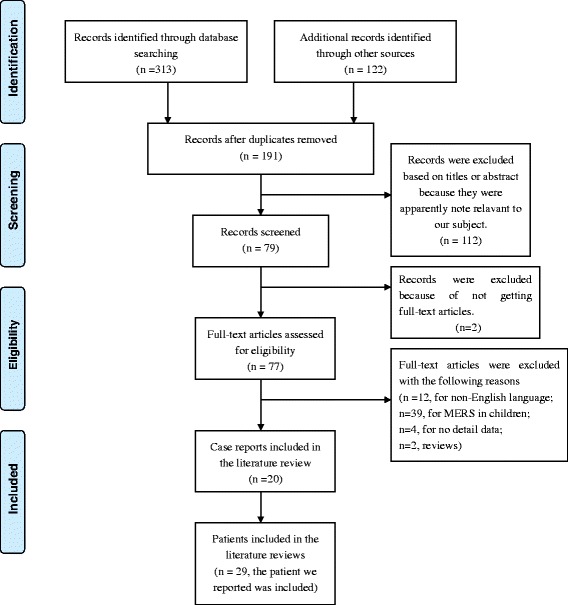
Flow diagram of studies selection process

A total of 435 articles between Januarary 2004 and March 2016 were identified by preliminary electronic literature search and hand search. The selection process was presented in Fig. 3. The characteristics of the included cases were presented in Table 1 and Table 2.

**Table 1:** Information of 29 adult-onset MERS cases

Reported by, location, reference	Case no.	Sex, age (years)	symptomsis	Neurological examination	Etiology
Beijing Chaoyang Hospital, China	1	M, 37	Fever, UT	cervical rigidity (+)	NQ
Tada et al. [1] Japan	2	F, 59	Fever, vertigo, lethargy	NS	NQ
	3	F, 18	Fever, seizure, delirium	NS	NQ
	4	M, 19	Fever, cough, delirium, seizure	NS	NQ
	5	F, 25	Fever, vesicular, headache, drowsiness, nausea	NS	VZV
	6	M, 22	Fever, hallucination, delirium	NS	NQ
Jun-ichi et al. [22] Japan	7	M, 31	Headache, fever, drowsiness, disorientation, memory disturbance	drowsiness, disorientation, memory disturbance	NQ
Jeong-Seon et al. [6] Korea	8	M, 59	Dysarthria, drowsiness,fever	Normal	NQ
Nida Tascilar et al. [27] Turkey	9	F, 26	Fever, headache, phonophobia, photophobia, dizziness, UT	Neck stiffness (+), positive Kernig’s sign, right truncal and gait ataxia	NQ
Makiko et al. [28] Japan	10	F, 23	Fever, headache, UT	Unsteady gait, patellar tendon reflexes, plantar reflexes, abdominal wall reflexes diminished	NQ
Henning Vollmann et al. [29] Germany	11	M, 42	Fever, vomit, headache	Mild ataxia, disturbance of gait	Tick-bites
Dimitri Renard et al. [30] France	12	M, 43	Stuporous state	GCS E4M5V2, mutism, persistent hiccup	Anti-Yo rhombenceh- halitis
Shingo Mitraki et al. [31] Japan	13	M, 29	Consciousness disturbance	Drowsy, disoriented	pneumonia
Hideki Shibuya et al. [10] Japan	14	M, 30	Fever, consciousness disturbance	Glasgrow coma scale: E4V1M6	Mycoplasma- pneumoniae
Balasubramanyam Shankar et al. [32] India	15	F, 28	Fever, vomiting, paresthesia	Drowsy, neck rigidity, up going plantar reflex	NQ
Soon Young Ko et al. [33] Korea	16	M, 30	Fever, alalia	dysarthra	Nonfulminant hepatitis A
Makoto Hibino et al. [34] Japan	17	F, 24	Fever, diarrhea, abdominal pain, weakness of right upper extremity	Right-side hemiparesis, hemianesthesia, Chaddock (+)	adenovirus
Yuji Tomizawa et al. [11] Japan	18	M, 49	Fever, gait difficulty	Wide-based gait, fine postural tremors, mildly exaggerate deep tendon reflexes	Legionella pneumophila- serogroup 2
Robert M et al. [35] America	19	M, 41	Fever, headache, delirium, consciousness disturbance, tremor, gait instability, paresthesias, UT	Slow thought, mild difficulty finding words, intention tremor of the left arm, dysmetria of lower extremities, broad-based gait	ipilimumba
Jing Jing Pan et al. [36] China	20	F, 18	Fever	(−)	NQ
	21	M, 26	Fever, acute UT	Nuchal rigidity (+)	NQ
	22	F, 23	Fever, headache, disturbance of consciousness	Kerning (+)	C-section
	23	M, 21	Fever, headache, acute UT, intestinal obstruction	Kerning (+)	NQ
Shuo Zhang et al. [37] China	24	F, 26	Headache, fever, seizure, somnolence	Somnolence	Mycoplasm-a
	25	F, 34	Dizziness, fever, somnolence	Somnolence	Mumps virus
	26	M,25	Headache, fever, cognitive impairment, behavioral disorders, confusion	Cognitive impairment, behavioral disorders, confusion	Herpes simplex virus
Eylem Degirmenci et al. [38] Turkey	27	M, 27	Headache, apathy, nausea, vomiting	Bilateral papilledema, mildly altered mental status	NQ
Naila Alakbarvova et al. [39] Turkey	28	M, 46	Headache, vomiting, nausea, diarrea, abdominal pain, generalized tonic-clonic seizure	Confusing with time disorientation	Amanita phalloides intoxication
Matthias Gawlitza et al. [40] Germany	29	F, 28	disorientated	Disorientated, confusion	Hemolytic uremic syndrome

*M* male, *F* female, *UT* urinary retention, *NS* no statement, *NQ* no required, *VZV* varicella zoster virus

**Table 2:** Auxiliary examination and treatment of 29 adult-onset MERS cases

Case no.	Initial examination						Treatment
	WBC (10^9^/L)	CRP (g/L)	Serum sodium (mmol/L)	CSF WBC (10^6^/L)	MRI	EEG	
1	10.99	9.41	131.8 (hyponatremia)	97	SCC	NE	mannitol, low dose methylprednisolone, ACV, anti-tuberculosis
2	NS	NS	NS	500	SCC	Slow BA	ACV, antibiotics
3	NS	NS	NS	17	SCC	Slow BA and spikes	PB, PSL
4	NS	NS	NS	Normal	SCC	Slow BA	ACV, PHT, PSL
5	NS	NS	NS	NE	SCC	NE	ACV
6	NS	NS	NS	Normal	SCC	Slow BA	ACV, antibiotics, PSL
7	18.8	17.0	NS	253	Entire CC and peripheral WM	NS	antibiotics
8	normal	normal	normal	normal	SCC and frontal WM	NS	No specific therapy
9	normal	normal	normal	408	SCC	normal	Ceftriaxone, ACV, ampicillin, catheterization
10	normal	normal	normal	normal	SCC, WM	NS	Methylprednisolone pulse, PSL, catheterization
11	NS	normal	132 (hyponatremia)	33	SCC	NS	Ceftriaxone, ACV, symptomatic therapy of headache and fever
12	NS	normal	133 (hyponatremia)	6	SCC, frontoparie-tal WM, putamina, thalami	Diffuse slowing wave	Antiepileptic treatment, methylprednisolone pulse, immunoglobulin treatment
13	elevated	elevated	NS	normal	Entire CC	NS	methylprednisolone pulse, immunoglobulin treatment
14	NS	NS	NS	NS	SCC	NS	Levofloxacin
15	NS	NS	NS	60	SCC, bilateral WM	Slow activity, frontal sharp wave	Empirical corticosteroids and ACV
16	3.48	NS	138	NS	SCC	abnormal	hemodialysis
17	normal	12.21	normal	NE	SCC	NS	No specific therapy
18	elevated	22.9	130	3	SCC	NS	Antibiotics
19	normal	normal	normal	128	SCC	NS	oral PSL and methylprednisolone pulse
20	6.08	normal	137.0	90	SCC	abnormal	methylprednisolone pulse, oral PSL
21	7.10	normal	130.2	100	SCC, insula, caudate nucleus	abnormal	Oral methylprednisolone and PSL
22	11.20	normal	138.6	12	SCC	abnormal	Oral methylprednisolone and PSL
23	8.20	normal	126.5	80	SCC	abnormal	Oral PSL
24	NS	NS	NS	3	SCC	Occipital slow waves	Mannitol, diazepam, macrolides antibiotics and moxifloxacin
25	NS	NS	NS	7	SCC	Occipital slow waves	Interferon, ribavirin
26	NS	NS	NS	112	SCC	Occipital slow waves	Ganciclovir, mannitol, antibiotics
27	normal	normal	normal	150	SCC	NS	Acetazolamide, antibiotics, oseltamivir
28	17.8	NQ	normal	normal	SCC	NS	Risperidone, clozapine, venlafaxine and diazepam, antipsychotic and intoxication treatment
29	NS	NS	NS	NS	SCC	NS	eculizumab

*WBC* white blood cell, *CRP* C-reactive protein, *CSF* cerebrospinal fluid, *EEG* electroencephalography, *NE* no examined, *NS* no statement, *BA* basic activity, *ACV* acyclovir, *PB* Phenobarbital, *PSL* prednisolone, *PHT* phenytoin, *WM* white matters

Of the 29 adult-onset MERS patients, 11 were from Japan, 8 from China, 3 from Turkey, 2 from Germany, 2 from Korea, 1 from France, 1 from India and 1 from America. From a geographical point of view, 86.2% of the countries were in Asia (25/29), especially in Japan (11/29).

The age of onset varied between 18 and 59 years old, with an average of 31. Twelve patients were females (41.38%) with a 12:17 female-to-male ratio. Fifteen patients had identified causes, including 5 virus infections, 3 pneumoniae, and 1 mycoplasma infection. One patient developed MERS due to Amanita phalloides toxication, one because of tick-bites. One patient had emotional and behavioral changes presenting with auditory hallucinations within 10 days after C-section.

Fever had preceded or simultaneously presented with neurologic symptoms in 24 patients. Twelve patients complained of headache while having MERS, and disturbance of consciousness was observed in 15 cases. Seizure occurred in 4 cases, and acute urinary retention in 6 patients. 75.9% of the patients (22/29) had an isolated lesion in the splenium of the corpus callosum. Six patients had lesions in both splenium and extracallosal. One patient had lesions in the entire corpus callosum. Lumbar punctures were performed in 23 patients, 15 of which had elevated CSF WBCs. Sixteen patients had their serum sodium reported, 6 of which had decreased levels. EEG was performed in 23 patients, 14 of which were abnormal.

The patients were treated with antiviral therapy, antibiotics, corticosteroids, IVIG, intravenous osmotic diuretic and isotonic fluid. Thirteen patients received corticosteroids therapy, 5 of which received a methylprednisolone pulse therapy. No case resulted in neurological sequelae.

## Discussion

We reported a previously healthy 37-year-old man who suffered MERS associated with acute urinary retention. A lesion in the SCC resulting in acute urinary retention has rarely been reported. We considered acute disseminated encephalomyelitis (ADEM) being the main differential diagnosis. In comparison with the lesions in MERS which show no contrast enhancement and usually disappear quickly [1], the corpus callosum lesions in ADEM are usually asymmetrical, contrast-enhancing, extend to the white matter and spinal cord [16], and resolve over weeks to months. Our patient’s cranial MRI showed an isolated abnormal signal in the SCC with no contrast enhancement. His spinal cord MRI showed no obvious abnormalities. The follow-up MRI scan revealed normalized findings within two weeks. So the patient was diagnosed as MERS instead of ADEM.

At first, a reversible isolated SCC lesion on MRI was diagnosed as MERS [1]. Recent studies suggested additional similar lesions in the cerebral white matter and anterior aspects of the corpus callosum in some encephalitis/encephalopathy patients should also been regarded as MERS (type 2 MERS) [4, 5]. Since the radiologic range of MERS had been expanded, patient no.7, 8, 10, 12, 15, 21 and 29 were included in the literature review.

Similar to child-onset MERS, most adult-onset MERS patients were also reported in Asia, including Japan, China and India. Interestingly, the majority cases were reported in recent five years. The phenomenon may be related to ethnics and social factors, as well as lack of diagnostic awareness and criteria before 2011. The common neurological manifestations of MERS in adult were headache and disturbance of consciousness. However, disturbance of consciousness and seizures were the most common neurological symptoms in children [15]. We suspect that it is related to children’s immature central nervous system and blood brain barrier.

The pathogenesis of MERS is still unknown. There are several hypotheses, including intramyelinic edema, axonal damage, hyponatemia, and oxidative stress [1, 17, 18]. High signal intensity on DWI and decreased ADC values of white matter have been observed in MERS. The possible explanation for this is intrmyelinic edema resulting from separation of myelin layers [19, 20] and local infiltration of inflammatory cells [1, 3]. In this review, we found that more than half (15/23) cases had elevated white cells in the CSF. A previous small sample study reported that patients with MERS has an elevated IL-6 and IL-10 levels in CSF, however, the sample is not enough for any conclusions to be drawn [17]. ADC may return to normal within a week if the intramyelinic edema or inflammatory infiltrate resolves quickly. Takanashi et al. [21] reported that most patients with MERS had mild hyponatremia with a mean serum sodium level (131.0 ± 4.1 mmol/L) lower than that of the healthy group. Our review revealed that 6/16 MERS patients had hyponatremia upon admission. All these indicate that hyponatremia might be a possible cause of MERS. Taken all together, MERS is a rare syndrome with unclear pathogenesis. None of the existing hypotheses explains why MERS specially involves the site splenium.

In any patients presenting with symptoms of encephalitis/encephalopathy who are found to have lesions in the white matter, ADEM should be included in the differentials [1, 4, 22, 23]. ADEM is a post-infectious inflammatory disorder which can present with seizures, focal neurological signs or altered mental status days to weeks after the presumed infections [24]. MRI with contrast shows various enhancements of the lesions in ADEM depending on the stages of the acuity [24]. Other differential diagnoses include posterior reversible encephalopathy syndrome (usually hypertension-related and has subcortical white matter lesion), multiple sclerosis (characteristic relapsing-remitting course), Marchiafava-Bignami disease (often seen in alcoholism), ischemia (usually irreversible and has vascular territory distributions), diffuse axonal injury (head trauma-related), lymphoma (positive contrast enhancement), and extrapontine myelinolysis (happens with electrolyte abnormality) [25].

Even though the evidence of methylprednisolone pulse therapy and IVIG’s efficacy on MERS is still lacking, they are recommended for patients with infectious encephalopathy regardless of the pathogen or clinicl-radiological syndromes [26]. In this review, only five MERS patients were treated with methylprednisolone pulse therapy and two with IVIG treatment. However, all patients without methylprednisolone pulse therapy or IVIG recovered clinically completely, which suggests that those treatments may not be necessary.

## Conclusion

In conclusion, we reported a case of an adult-onset MERS with acute urinary retention. Taken together with the previously reported cases, we suggest that MERS in adults is an entity with a broad clinico-radiological spectrum and the prognosis is good. From a geographical point of view, most adult-onset MERS patients were also reported in Asia. The common neurological manifestations were headache and disturbance of consciousness. There are similar characteristics between MERS in adults and children, also some differences.

## Abbreviations

ADC: Apparent diffusion coefficient
ADEM: Acute disseminated encephalomyelitis
CSF: Cerebrospinal fluid
EEG: Electroencephalogram
MERS: Mild encephalitis/encephalopathy with reversible splenial lesion
SCC: Splenium of the corpus callosum
